# Cincinnati Sanitarium

**Published:** 1882-08-20

**Authors:** 


CINCINNATI SANITARIUM.—See the advertisement of this excellent institution in this Journal.

